# Mechanical Force Acting on Ferrogel in a Non-Uniform Magnetic Field: Measurements and Modeling

**DOI:** 10.3390/mi13081165

**Published:** 2022-07-23

**Authors:** Felix A. Blyakhman, Alexander P. Safronov, Andrey Yu. Zubarev, Grigory Yu. Melnikov, Sergey Yu. Sokolov, Aitor Larrañaga Varga, Galina V. Kurlyandskaya

**Affiliations:** 1 Department of Biomedical Physics and Engineering, Ural State Medical University, Ekaterinburg 620028, Russia; feliks.blyakhman@urfu.ru (F.A.B.); sergey.sokolov@urfu.ru (S.Y.S.); 2 Institute of Natural Sciences and Mathematics, Ural Federal University, Ekaterinburg 620002, Russia; alexander.safronov@urfu.ru (A.P.S.); a.j.zubarev@urfu.ru (A.Y.Z.); grigory.melnikov@urfu.ru (G.Y.M.); 3 Institute of Electrophysics UB RAS, Ekaterinburg 620016, Russia; 4 Advanced Research Facilities (SGIKER), Basque Country University UPV/EHU, 48940 Leioa, Spain; aitor.larranaga@ehu.eus; 5 Departamento de Electricidad y Electrónica, Universidad del País Vasco UPV/EHU, 48080 Bilbao, Spain

**Keywords:** magnetic particles, ferrogels, biomimetic materials, magnetic field, attractive force, modeling, biomedical applications

## Abstract

The development of magnetoactive microsystems for targeted drug delivery, magnetic biodetection, and replacement therapy is an important task of present day biomedical research. In this work, we experimentally studied the mechanical force acting in cylindrical ferrogel samples due to the application of a non-uniform magnetic field. A commercial microsystem is not available for this type of experimental study. Therefore, the original experimental setup for measuring the mechanical force on ferrogel in a non-uniform magnetic field was designed, calibrated, and tested. An external magnetic field was provided by an electromagnet. The maximum intensity at the surface of the electromagnet was 39.8 kA/m and it linearly decreased within 10 mm distance from the magnet. The Ferrogel samples were based on a double networking polymeric structure which included a chemical network of polyacrylamide and a physical network of natural polysaccharide guar. Magnetite particles, 0.25 micron in diameter, were embedded in the hydrogel structure, up to 24% by weight. The forces of attraction between an electromagnet and cylindrical ferrogel samples, 9 mm in height and 13 mm in diameter, increased with field intensity and the concentration of magnetic particles, and varied within 0.1–30 mN. The model provided a fair evaluation of the mechanical forces that emerged in ferrogel samples placed in a non-uniform magnetic field and proved to be useful for predicting the deformation of ferrogels in practical bioengineering applications.

## 1. Introduction

Ferrogels (FGs) are novel advanced responsive soft composites [1,2], which are extensively studied for their promising applications in biomedicine and biotechnologies [3,4,5,6,7]. A ferrogel is a composite polymeric material that chemically contains a loosely cross-linked polymeric network evenly distributed in a liquid medium, usually water, and magnetic particles embedded into this network [8,9]. The volume and the shape of the gel polymeric network, in general, can change, to a large extent, due to the application of any external mechanical force and likewise due to a variety of external non-mechanical stimuli such as the molecular and/or ionic compositions of the liquid medium, temperature, pH, etc. [10].

In addition, in the case of FGs, there is a possibility to control their physical properties and location by the remote action of an external magnetic field due to the presence of the embedded magnetic particles employed as a filler [11]. Magnetic ferrogels are of special interest as model magnetic materials that mimick the properties of natural tissues [4]. Hydrogel represents a model tissue and ferrogel represents the tissue with embedded magnetic particles. Magnetic particles in an external magnetic field create stray fields. Their analysis can be used for the visualization of the geometry of a tumor or to simply define the appropriate moment to start chemotherapy or mechanotherapy based on the particle concentration [12,13]. Such an analysis can be performed using data from magnetic field sensors combined with a scanning system. This means that in each device there are at least two magnetic materials working together: the magnetic field sensitive element and magnetic particles [14,15]. Each particular application requires careful analysis of the work parameters of the magnetic sensitive element and particles working in one device. Each material has its own field interval for which it has high sensitivity with respect to the applied magnetic field (sensitive element) or high/detectable magnetic moment value; both field intervals must have an overlap zone for the operation of the detection regime [4].

In bioengineering, FGs have been extensively studied as responsive working bodies in various detectors and actuators that provide transfer of a mechanical force under remote control of a magnetic field [16,17,18,19,20]. In addition, spherical FG microparticles can be used for magnetic biosensing and for drug delivery purposes, in which the force associated with each particle as compared with the forces associated with corresponding biochemical bonds are also very important [4,21]. Furthermore, the general biocompatibility of FGs provides versatile options of their prospective usage in medicine as magnetoactive matrixes for targeted drug delivery and replacement therapy [22,23,24].

The desired functional properties of FGs are based on magneto-mechanical coupling, which occurs if the magnetically active elastic material interacts with an applied magnetic field. Therefore, theoretical and experimental consideration of the mechanical force that appears in ferrogel placed in a magnetic field is highly appropriate concerning the development of bioengineering systems and devices.

Theoretical considerations have mostly been developed for the influence of a uniform magnetic field on the shape and elasticity of a ferrogel. It has been shown [25,26,27,28] that uniformly magnetized ferrogel usually exhibited positive field-induced magnetic strain in the uniform magnetic field, i.e., the ferrogel elongated in the direction along the axis of the field lines and simultaneously contracted in the transverse direction. At the same time, in terms of a more realistic model of separate magnetic particles dispersed in an elastic medium, it has been shown [29,30] that the induced deformation might also be negative, exhibiting shortening in the direction along the field lines and swelling in the transverse direction. The switch between these alternatives is governed by the way in which magnetic particles are distributed in the elastic matrix.

Experimental studies of the magneto-deformation phenomenon in a uniform magnetic field have shown a variety of scenarios for the mechanical response of a ferrogel to the application of an external magnetic field. Examples of both positive and negative field-induced magnetic deformation have been reported [31,32]. The typical feature has been a substantial change in the volume of ferrogel in an applied magnetic field. Ferrogel in an equilibrium with liquid medium is an open system and water molecules can freely cross its free surfaces entering or leaving the network, resulting in net swelling or contraction of the ferrogel sample in the applied magnetic field.

In the scientific literature, characterization of the behavior of ferrogels in non-uniform magnetic fields is much less extensive. There are few experimental studies [33,34] which present some particular examples of mechanical deformation of ferrogels under such a field. Meanwhile, a non-uniform magnetic field is more easily maintained as compared with a uniform field, and hence, the former is commonly more available in terms of practical applicability. In this regard, an understanding of ferrogel deformation is important for applications of magnetic field sensors in the detection of ensembles of magnetic particles embedded in natural tissue. The abovementioned particles can be both the carriers of drugs and the basis for thermal ablation or hyperthermia treatment. The procedures both include the application of an external field [35,36]. However, the precise definition of the time to start the therapy is related to the state in which magnetic particles form the “ferrogel-like” natural structure, and therefore, it is to understand its properties under application of an external field.

The objective of the present study was to measure directly the mechanical force which appeared by placing a ferrogel sample in a non-uniform magnetic field. Ferrogel samples based on the chemically cross-linked polyacrylamide with embedded submicron-sized magnetite particles were elaborated in this research and the experimental setup for measuring the mechanical force on ferrogel samples in a non-uniform magnetic field was designed, calibrated, and tested. The obtained experimental data were used to verify a theoretical model for evaluating this force as a function of field intensity and the content of magnetic particles in the ferrogel.

## 2. Materials and Methods

### 2.1. Magnetic Particles, Ferrogels, and Model Epoxy Composite Sample

**Figure 1:**
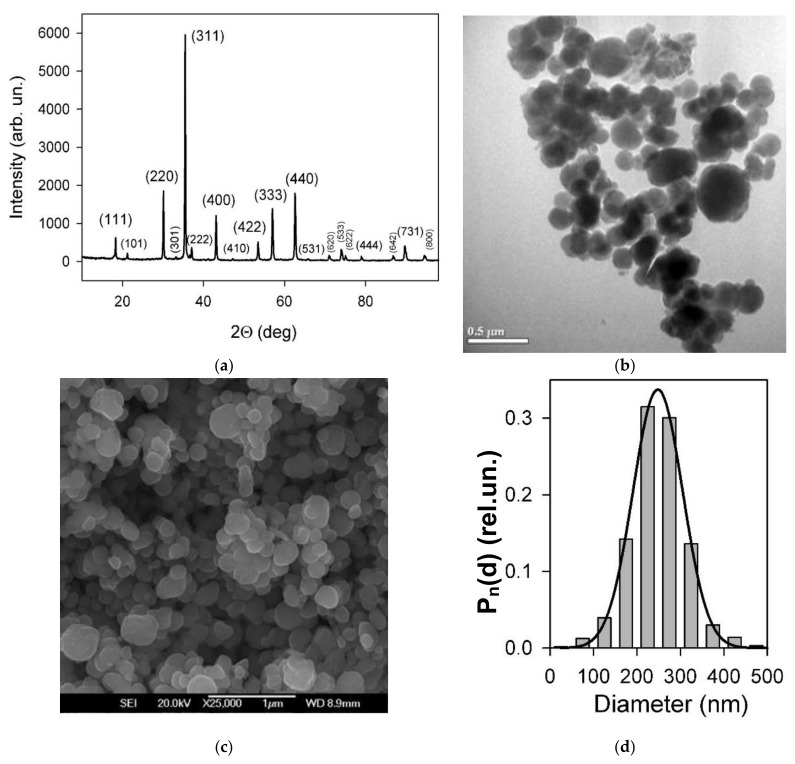
(**a**) XRD spectrum of magnetic particles with Miller indexes corresponding to each reflection; (**b**) transmission electron microscopy images; (**c**) scanning electron microscopy images, of magnetite particles used as a magnetic filler for ferrogels and model epoxy composite; (**d**) the average PSD for 632 particles, the line corresponds to the normal distribution probability function with a median of 248 nm and a dispersion of 57 nm.

Commercial magnetic microparticles (Alfa Aesar, Ward Hill, MA, USA) were used in the present study as a magnetic filler. The crystalline structure of particles was characterized by X-ray diffraction (XRD) using a Bruker D8 Discover (Bruker, Billerica, MA, USA) diffractometer operated at CuK_α_ radiation (wavelength λ = 1.5418 A) with a graphite monochromator and a scintillation detector. The diffractogram was refined using the Rietveld full-profile method (Figure 1a). The crystalline structure contained 94.6% magnetite (Fe_3_O_4_) and 5.4% goethite (FeO(OH)) phases.

Transmission electron microscopy (TEM) (JEOL JEM2100, Tokyo, Japan, microscope operating at 200 kV) and scanning electron microscopy (SEM) (JEOL JSM-7000 F, Tokyo, Japan) images of magnetite microparticles were provided for the sets of the particles (Figure 1b,c). The particles were close to spherical with caliper sizes from 50 to 400 nm. The particle size distribution was obtained by graphical analysis of 632 particle images using quasi-spherical approximation. For the analysis of TEM images (Figure 1), they were fitted using the normal (Gaussian) distribution function Pn(d) with a median of 248 nm and a dispersion of 57 nm. Typically, the PSDs in ensembles of particles obey the lognormal pattern. However, there can be exceptions, for example, particles that are synthesized using techniques such as precipitation and growth, or particles that are subjected to some pretreatments; for a specific batch of such commercial magnetic particles, the normal distribution function provides a much better fit than lognormal. Meanwhile, if using PSD, it should be noted that its validity is limited to the 50–500 nm range of the caliper size.

The ferrogel samples were synthesized using free-radical polymerization in 0.8 M water solution of monomer, i.e., acrylamide (AppliChem, Darmstadt, Germany). The networking was provided by a cross-linker, i.e., *N*,*N*′-methylene-bis-acrylamide (Merck, Schuchardt, Honenbrunn, Germany) in a 1:100 molar ratio to the monomer. Then, preweighted portions of commercial powdered magnetite with micron-sized particles were added to the reaction mixture. A polymeric thickener (natural polysaccharide guar, Sigma Aldrich, St. Louis, MO, USA) was added to the reaction mixture at a 0.56% concentration to prevent sedimentation of magnetite particles during the synthesis. The mixture was vigorously stirred, and then the initiator (ammonium persulfate) was added to the mixture with a 3 mM concentration. The polymerization occurred in polyethylene cylindrical tubes, 8 mm in diameter, and it started almost immediately at room temperature due to the catalytic performance of the traces of Fe(II) at the surface of magnetite particles. As the reaction mixtures lost their fluidity, the tubes were placed in a thermostat at 80 °C for 60 min to accomplish polymerization. After polymerization, the cylindrical ferrogel samples were taken out and washed in distilled water for two weeks with daily water renewal to eliminate traces of salts and monomers. The final contents of magnetite particles in the ferrogel samples equilibrated in water were determined by thermogravimetry using a NETZCH STA403 thermal analyzer (NETZSCH Geratebau Gmbh, Selb, Germany) via temperature scan up to 1000 °C at 10 K/min rate. The contents of magnetite in the series of ferrogel samples were: 4.3, 6.5, 16.7, and 23.8% by weight. To perform the mechanical measurements, the ferrogel samples were cut into cylindrical pieces, 9 mm in height and 13 mm in diameter. Note, the diameters of ferrogel samples were larger than that of the cylindrical tubes for the synthesis due to the swelling of ferrogels during the washing cycles.

The model composite sample (KDA epoxy resin (Chimex Ltd., St. Petersburg, Russia, as a polymer matrix) was a sphere, 4 mm in diameter. The resin was mixed in a 6:1 weight ratio with a tri(ethyl)-tetra(amine) hardener (Epital, Moscow, Russia). The particles of the filler were from the same batch as the ferrogel samples. They were mixed with liquid resin at 25 °C to obtain a homogeneous mixture, followed by curing. The composite sample was prepared with a magnetic particle concentration of 30.1% by weight.

Magnetic characterization was conducted using a vibrating sample magnetometer (VSM) (Cryogenics, Ltd., London, UK) at room temperature. The magnetic hysteresis loops of the magnetic particles, gel, ferrogel and composite samples were measured in a polycarbonate capsule. For the gel and ferrogel sampels, we followed a specially developed protocol [32] in order to control their mass. The primary magnetization curve of the composite sample was measured for the low field interval of linear dependence of M(H), where M is the magnetization and H is the applied external field.

### 2.2. Measurement of Magnetic Force on Ferrogel in a Magnetic Field

**Figure 2:**
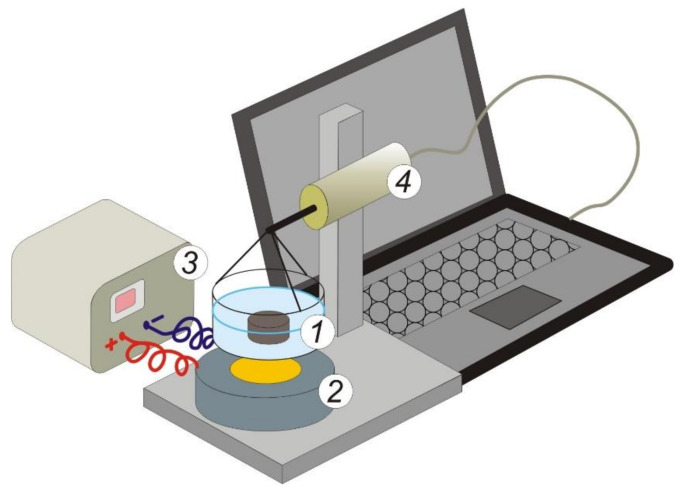
Schematic sketch of the experimental setup for measuring the mechanical force on ferrogel in a non-uniform magnetic field. (**1**) Cuvette with a ferrogel sample; (**2**) electromagnet; (**3**) stabilized power supply; (**4**) force transducer. See explanation in the text.

The experimental setup (Figure 2) for measuring the mechanical force on ferrogel in a non-uniform magnetic field was built around a massive tripod with a force transducer fixed on it. A thin-walled polystyrene cuvette filled with distilled water and containing a ferrogel sample was mechanically connected to the force transducer using rigid copper braces. A commercial electromagnet (EM) CL-34/18 (Cinlin, Guangzhou, China) was placed under the cuvette coaxially with the sensitive lever of the force transducer and the cuvette at a distance of 0.5 mm from the surface of the EM core (18 mm in diameter). To avoid the impact of the EM on the metallic lever of the sensor, the distance between the EM and the lever was set at 8 cm.

The force transducer was designed with a force sensor and operational amplifier. A commercial C05 force sensor (VIP, Yekaterinburg, Russia) based on silicon strain gauges on a sapphire membrane was used; according to the manufacturer specifications, the sensor lever displacement at a maximum load of 5 N was 0.25 mm and the output signal at a maximum load was 250 mV. After amplifying the signal, the dynamic range of the force transducer was limited to 0.2 N, and the force measurement accuracy (output RMS value of the noise) was 0.03 mN. The force transducer was calibrated with the use a set of weights for analytical balance.

The magnetic field strength of the EM was provided by using a stabilized power supply PS-1502D (Element, Shenzhen, China). The voltage to the EM coil (25.8 Ohm) was set discretely in the range from 0 to 15 V with a step of 1 V. The characteristics of the magnetic field produced in the electromagnet were determined experimentally using a MPU gaussmeter (“Centre”, Moscow, Russia; State register number of approved type measuring instruments 28134-04). The systematic error was evaluated in accordance with gaussmeter metrology characteristics.

Control experiments with the cuvette filled with water were conducted to confirm the effect of the EM on the force transducer. No interaction between the EM and the metallic lever of the sensor was found in full range of the magnetic field strength. Thus, the voltage value recorded at the output of the force transducer served as a measure of the forces of attraction of ferrogel samples to a magnetic field source.

## 3. Results and Discussion

**Figure 3:**
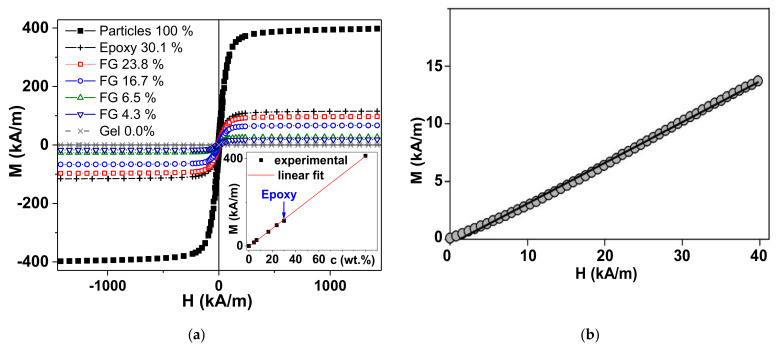
Magnetic hysteresis loops of magnetite particles in the VSM: (**a**) Magnetite particles (Particles 100%), ferrogels with different concentration of the magnetite particles used as a filler (FG 23.8%, FG 16.7%, FG 6.5%, FG 4.3%, Gel 0.0%—the numbers correspond to the weight percent of the particles), and epoxy composite with 30.1 wt.% of the particles. Inset shows the concentration dependence of the magnetic moment of the samples of different types mentioned above for the magnetization measured in the external field of 1450 kA/m, being close to saturation magnetization. Points are experimental data, a line is the linear fit, the arrow indicates the point corresponding to model epoxy composite; (**b**) the low-field dependence of the initial magnetization (primary magnetization curve) of the model epoxy sample (sphere) with 30% (wt.) of magnetite particles, measurement performed starting with the demagnetized state. The solid lines are guides for observations.

The magnetic hysteresis loops M(H) of all the used samples are shown in Figure 3a. As expected, the highest value of the saturation magnetization corresponds to the magnetic particles of the filler, shown in Figure 3 as “Particles 100%”. According to it, the saturation magnetization (Ms) was found to be as high as M_s_ = 390 ± 10 kA/m, the remnant magnetization was M_r_ = 30 ± 1 kA/m, and the coercivity was H_C_ = 6.2 ± 0.1 kA/m. These values are close to the typical values of the corresponding parameters for magnetite particles of the size under consideration. This means that the particles were reasonably magnetically soft in the typical range for this type of material [37]. For simplicity, we assigned the value of saturation magnetization (Ms) for the magnetization measured in the external field of 1450 kA/m. Figure 3a shows that such an assumption is reasonable. The Ms values of the ferrogel samples show a linear dependence on the magnetic filler concentration.

Although it is expected for materials with a low concentration of MPs [12,32], in the case under study, both the Ms of the particles themselves and the epoxy composite are reasonably fit by the same linear dependence. This is further described in the discussion of the theoretical model. The last observation is very important when considering substituting the measurements of the initial magnetic permeability using ferrogels which do not allow precise control of the shape and the weight. Figure 3b shows the primary magnetization curve of the magnetic particles forming part of the epoxy composite, with the features also confirming the approximation of the spherical shape. The conclusion can be made in accordance with the standard procedure of a simple evaluation of the shape of the magnetic particles, as previously shown (the effective susceptibility is controlled by the demagnetizing factor) [38].

**Figure 4:**
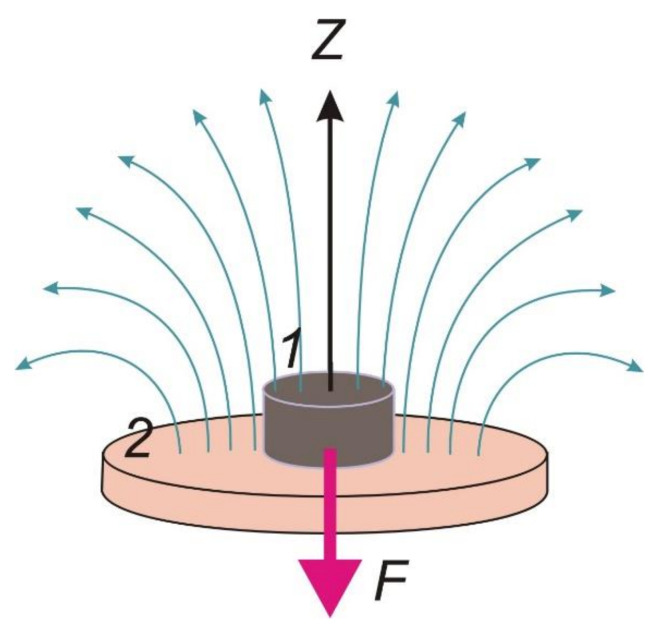
General sketch of the geometry of the considered measuring system: *1*—ferrogel sample; *2*—electromagnet. Small blue arrows illustrate the distribution of the magnetic field lines and the big magenta arrow shows the direction of the force F orientation.

**Figure 5:**
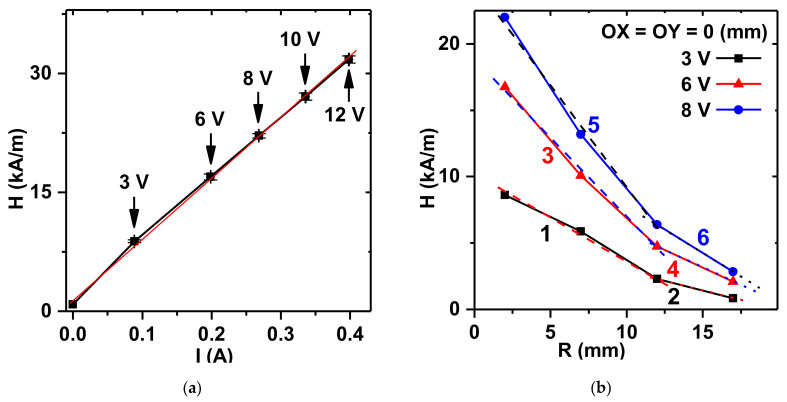
Characteristic plots of the field strength of the electromagnet: (**a**) Dependence of the field in the center of the surface of the magnet on the electric current value. H(I) dependence can be fitted by the equation y = 76.6X + 1.5; (**b**) dependences of the field strength on the distance from the surface. Dependences can be fitted by the following equations: 1 − y = 0.6X + 10.0, 2 − y = 0.3X + 5.8, 3 − y = 1.2X + 18.9, 4 − y = 0.5X + 11.0, 5 − y = 1.6X + 24.8, and −y = 0.7X + 14.9.

A general sketch of the system for the FG force measurements in a non-uniform magnetic field is shown in Figure 4. It was specially designed, calibrated, and tested in the present study. Figure 5a shows the experimental results for calibration of the values of the intensity of a magnetic field in the center of the surface of the magnet, fed the electric current from the current supply. The plot is linear and it provides control over the constant magnetic field strength.

Figure 5b presents the dependence of the field strength with respect to the distance between the surface of the magnet and the point under consideration over the central point of the magnet. The field strength decays rapidly as the distance increases. Within 10 mm from the surface of the magnet, the dependence is close to linear. Although the slopes are different for different levels of electric current, their simple transformation yields a universal dependence on field strength (*H*) within 10 mm distance from the magnet:

*H* = *H*_0_(1 − 0.073*z*) (1)

where *H*_0_ is the field intensity at the surface of the magnet, and *z* is the distance from the surface of the magnet in mm.

Table 1 shows the values of the force of attraction measured in FG samples with different concentrations of magnetic particles at different levels of voltage on the electromagnet coil, which provided the different intensities of a magnetic field as shown in Figure 5a. Selected data are given, more detailed data is presented below. One can see that the emerged mechanical forces were in the milli-Newton range and varied from the lowest value of approximately 0.1 mN to approximately 30 mN. At a given concentration of magnetic particles in the FG, the higher the intensity of the magnetic field, the stronger the force of attraction. In addition, at any value of the applied field, the fkorce of attraction increased with increased concentration of magnetic particles in the FG samples.

### 3.1. Theoretical Model

A rigorous theoretical evaluation of the force of attraction of a magnetizable sample to a magnet presents a very cumbersome mathematical problem. In addition, this problem is difficult due to the uncertainness in the spatial distribution of particles inside a gel and also due to the effect of their magnetic interaction on the macroscopic magnetic characteristics of a composite. The known microscopic theories of magnetic properties of magnetic composites deal either with the spatial homogeneous distribution of particles or with composites with internal heterogeneous structures (for example, with chain-like aggregates). Therefore, in this study, we consider the susceptibility of a ferrogel sample as an empirical parameter.

The layout of the model is presented in Figure 4. A cylindrical ferrogel sample (1) is placed on the flat surface of the electromagnet (2) so that the axis of the sample and the field lines of the magnet are parallel. We introduce the coordinate axis *z* aligned along their mutual symmetry axis. The diameter of the sample is assumed to be less than the diameter of the solenoid. Therefore, the tangential dependence of the field in the direction perpendicular to the axis *z* was systematically neglected.

The absolute value of the force acting on the ferrogel can be calculated as follows [35]:

(2) $$F=\mu_{0}\left|\int M\frac{dH}{dz}dV\right|$$

where $\mu_{0}$ is the magnetic permeability of vacuum; *M* is the sample magnetization; and *H* is the magnetic field, created by the electromagnet in the space where the ferrogel is placed. 

The integration was done over the sample volume *V*. The problem of determining the magnetization *M* inside a magnetizable particle is solved analytically only for an ellipsoid placed in a homogeneous external field (see, for example, [39]). For cylindrical samples, even in a uniform field, any results are unknown, needless to say, for the problem of a cylinder in inhomogeneous field, which can be solved only numerically and requires large computer resources and time-consuming calculations. Therefore, analytical theoretical modeling can only be done based on certain simplifications. Here, we present a simplified approach, which neglects the details of the interior distribution of magnetic particles and considers ferrogel as a uniform magnetizable material.

Here, to obtain the results for the force (2) in a visible mathematical form, with reasonable estimates, we assume that *M* is uniform across the ferrogel cylinder and equals the magnetization of the ferrogel in a uniform field averaged over the cylindrical sample (see Figure 5). Next, we neglect magnetic field-induced deformation, since, as observations show, these deformations of the sample are very small. In these approximations, the average field can be calculated according to the following equation:

(3) $$\bar{H}=\frac{1}{h}\int_{z_{2}}^{z_{1}}Hdz$$

where *z*_1_ and *z*_2_ are the coordinates of the top and the bottom of the cylinder, and *h* is its height. 

Taking the linear diminishing of the field along the height of the ferrogel (see Figure 4 and Equation (1)), the average field reads are rather simple as:

(4) $$\bar{H}=\frac{H_{1}+H_{2}}{2}$$

where *H*_1_ and *H*_2_ stand for the field intensity at the upper and bottom faces of the ferrogel cylinder (Figure 4).

Then, the magnetization of the ferrogel is as follows:

(5) $$M=\frac{\chi }{1+N\chi }\frac{H_{1}+H_{2}}{2}$$

where *χ* is the magnetic susceptibility and *N* stands for the demagnetization factor. This factor was evaluated and tabulated in [40], where it was found to be approximately 0.43 for the cylindrical sample with 9/13 height-to-diameter ratio similar to that elaborated in the present study.

Introducing Equation (5) into Equation (2), after integration we, finally, obtain the following equation for the dependence of the mechanical force on the field intensity:

(6) $$F=\mu_{0}\frac{\chi }{1+N\chi }\frac{V}{h}\frac{H_{2}^{2}-H_{1}^{2}}{2}$$

Note that Equation (6) relates to a non-uniform magnetic field, although it is characterized by an average field intensity along the height of the cylinder according to Equation (4). The difference in the numerator in Equation (6) originates from the gradient of a non-uniform field at normal direction to the magnet.

### 3.2. Verification of the Model

To apply Equation (6) to the experimental values of the mechanical force, the estimation of the magnetic susceptibility (*χ*) must to be provided. It is, however, not straightforward and meets experimental difficulties mostly due to the fact that the ferrogel samples are open systems in an equilibrium with a supernatant water phase. Conventional instruments such as vibrating sample or SQUID magnetometers are not well suited for this purpose because of the rapid change in the mass of the sample. In our previous works we developed some protocols for measurements with ferrogel samples, including the definition of the change in the mass as a function of time [41]. However, in each particular case, the rate of the change in mass depends on a number of parameters such as the type of the gel matrix, and size/shape of the FG sample. There are other examples of laboratory equipment designed specifically for such measurements [42], but they address the susceptibility in oscillating fields rather than the quasi-static values. Therefore, in this study, we evaluated the susceptibility of ferrogel samples using a model epoxy composite filled with the same magnetite particles in 30% weight fraction. The mixing of the composite was performed using the same method as the preparation of the reaction mixtures for the ferrogel sampes and it was assumed that the distribution of magnetic particles in the epoxy composite was close to that in the ferrogel samples.

Figure 3b shows the plot for the initial magnetization of the model composite in low-field range provided by the electromagnet of the laboratory setup in Figure 4. The plot M–H is linear and its slope can be obtained as follows:

(7) $$M=\frac{\chi }{1+N\chi }H$$

Here, the demagnetization factor *N* equals 1/3, since the sample is a sphere.

Equation (7) was used to calculate the volume susceptibility for the model sample. Based on the assumption that the distribution of the particles in the model epoxy composite is the same as in the ferrogel sampes, we calculated the susceptibility of the ferrogel sampes proportional to the volume fraction of particles:

(8) $$\chi_{FG}=\chi_{EC}\frac{\varphi_{FG}}{\varphi_{EC}}$$

where *χ_FG_* is the susceptibility of the ferrogel; *χ_EC_* is the susceptibility of the epoxy composite; *φ_FG_* and *φ_EC_* are the volume fractions of magnetite particles in ferrogel and the epoxy composite, respectively. Table 2 shows the obtained numerical values.

It is noticeable that the susceptibility values are close to the slope for M–H dependence, as expected for spherical particles [43,44]. At the highest volume fraction of particles, i.e., 9.57% in the case of the epoxy composite, the slope of M–H dependence is lower than the susceptibility by approximately 10% of the value. At low content of magnetite particles, susceptibility is almost equal to the M–H slope.

The values presented in Table 1 were used for the evaluation of the mechanical force acting in ferrogel sampes at different field intensities (*H*_0_). It is noteworthy to emphasize that the field is non-uniform, and the field intensity *H*_0_ corresponds to the surface of the electromagnet and diminishes with the distance from the surface, according to Equation (1).

**Figure 6:**
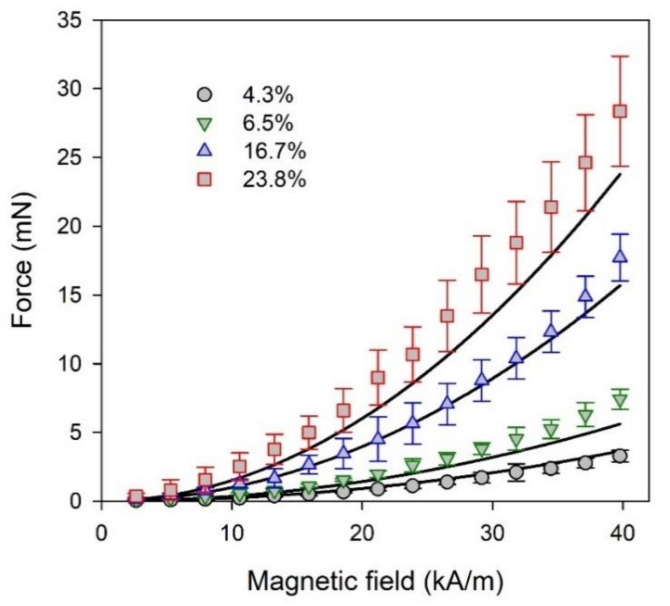
Mechanical forces emerged in ferrogel samples in a non-uniform magnetic field as a function of the field intensity at the surface of the electromagnet. Symbols correspond to the experimental values measured for the ferrogel sampes with different weight fraction of magnetite particles given in the legend. Lines are plots calculated using Equation (6).

The comparison of the actual forces measured using the experimental setup with the results of modeling are displayed in Figure 6. The dependence of the acting force on field intensity is parabolic, in accordance with Equation (6). The force systematically enhanced with the magnetite content in ferrogel. It is a presumable effect of an increase in magnetic load.

The calculated values of the acting mechanical force fit the experimental data within the limits of the experimental accuracy. The concurrence is very good in the case of ferrogel sampes with 4.3% and 16.7% weight fraction of magnetite particles, and not as such in the case of ferrogel samapes with 6.5% and 23.8% of magnetite. The deviation seems to be rather stochastic and might stem from the difference in the particle distribution in the interior of ferrogel samples, the uniformity of which it is difficult to maintain. It is also difficult to establish identical particle distribution in all the ferrogel sampes and in the model epoxy composite. The nature of these materials is different. It might certainly question the estimation of the susceptibility for ferrogel samples. However, our recent studies of the concentration dependence of saturation magnetization of epoxy composites for the same type of Fe_3_O_4_ magnetic particles indicated that the saturation magnetization linearly depended on their weight fraction in the composite in the concentration range from 1 to 30%. It happens despite the structural differences observed by the microscopy evaluation [45]. This means that our previous studies indicate that concentration is the most important parameter for this type of epoxy composite and their selection for the initial susceptibility evaluation is justified. Concerning all this, we consider the simplified model presented by Equation (6) to be in fair agreement with the experimental results.

The possible influence of the deformation of ferrogel on the emerged mechanical force should also be considered. Ferrogels are soft materials in the sense of their deformation ability. Their degree of deformation in an external magnetic field might be noticeable [6]. The influence of sample height on mechanical force within the applicability of the model can easily be obtained by combining Equations (1) and (6), which shows as a linear increase in mechanical force with diminishing height of the ferrogel cylinder, assuming its volume is constant. Meanwhile, the observed deformation for the ferrogel samples under study did not exceed 6% [6]. According to the linear dependence between height and force, the increase in the latter should also be the same. Such gain is much lower than the experimental error of the measurement for force which was about 10%. Thus, the influence of sample deformation under force can safely be neglected.

**Figure 7:**
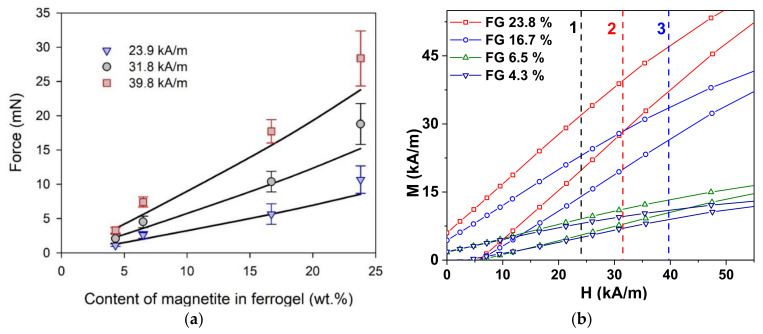
(**a**) Acting force as a function of magnetite weight fraction in ferrogel at selected levels (23.9, 31.8, and 39.8 kA/m) of magnetic field intensity at the surface of the electromagnet. Symbols correspond to the experimental values. Lines give the model evaluation for the dependence according to Equation (6); (**b**) parts of the magnetic hysteresis loops measured by VSM for ferrogel samples with 23.8, 16.7, 6.5, and 4.3 wt.% of magnetic filler. Dashed vertical lines indicate the same fields as used for part (**a**): 1—23.9 kA/m, 2—31.8 kA/m, and 3—39.8 kA/m. It is important to mention that the field strengths at the part (**a**) correspond to the gradient field intensity at the magnet surface. However, the field strengths at the part (**b**) correspond to the uniform external magnetic field used for VSM measurements.

Figure 7 shows a comparative result between the experimental and theoretical models for the dependence of force on the weight fraction of magnetite for selected levels of magnetic field intensity at the surface of the electromagnet. It is noticeable that the dependence of force on the weight fraction is linear within the range of particle content covered in the present study, both for the experimental values and for the theoretical prediction. In terms of the applied model, it is the consequence of Equation (8) which implies the proportionality of susceptibility on the volume fraction of magnetic particles in ferrogel. The linear dependence of the volume fraction stays very close to linear in the weight fraction as well in the range of low content of solid particles. Substantial deviation occurs at loads higher than 40–50% by weight.

Although we provided VSM results for all types of samples used in the present study, the measurements of the acting force were made in different conditions. The first difference is the type of magnetic field, which is a gradient in the force measurements and a constant in the case of VSM. The second difference is the value of the field strength. The analyzed saturation magnetizations are very important parameters for understanding the basic properties of magnetic materials and their effectiveness in biomedicine. However, usually, magnetic materials are not used in saturated states in applications, since a high magnetic field is costly, difficult to apply in microdevices, and it is controlled by special regulations [46].

Nevertheless, presentation of the results of VSM measurements for the scale corresponding to the typical magnetic field values used in acting force measurements seems to be useful for understanding the mechanical force results. Figure 7b shows that parts of the M(H) magnetic hysteresis loops correspond to a positive external magnetic field (both in decreasing and increasing magnetic fields) for all types of the ferrogels. In order to make such a comparison easier, we added vertical dashed lines corresponding to the field values selected (see Figure 7a) for experimental measurements and model calculations.

In the present work, we did not consider the possibility of increasing the magnetic moments of the ferrogel samples with zero applied field by previous application of the high constant external field. Such an action could result in an increase in the remnant magnetization and the magnetic moment of the sample by the application of a small magnetic field afterwards. At the same time, in previously magnetized samples, the initial magnetic susceptibility behavior can be quite different, and therefore, additional experimental and theoretical studies are needed in order to understand the mechanical force acting on the ferrogel in a non-uniform external magnetic field.

The successful use of FGs for biomedical applications critically depends on understanding the interaction mechanisms between the magnetic composites and a magnetic field, in particular, an understanding of how the driving force of the magnetic field depends on the physicochemical properties of the FGs. Therefore, we designed an original experimental setup to obtain direct measurements of the force of attraction of FG samples to a non-uniform magnetic field source.

Composite FGs with interpenetrating chemical (polyacrylamide) and physical (natural polysaccharide—guar) polymer networks with magnetic sub-microparticles of magnetite embedded in their structure were used as objects in this study. The choice of FG was based on the knowledge that gels with interpenetrating networks are biocompatible materials with mechanical properties close to those of biological soft tissues [47,48,49,50]. It is also known, that magnetite magnetic particles have low toxicity and are widely introduced for biomedical applications [51,52].

The obtained results could be useful for predicting the deformation of ferrogels in practical bioengineering applications. Given the modulus of a ferrogel, its deformation can be readily evaluated based on the model prediction for the force emerged. Owing to its simplicity, the consideration might be readily extended for more complicated setups of FG applications in biomedicine, for instance, controlled delivery of drugs and bioactive agents to tissues and organs, including the location and concentration of magnetic particles in natural tissues [53].

## 4. Conclusions

The experimental setup for measuring mechanical force on ferrogel samples in a non-uniform magnetic field was designed, calibrated, and tested. This methodological approach made it possible, for the first time, to perform direct measurements of the force of attraction of ferrogels (FGs) to the source of the non-uniform magnetic field. The values of the force of attraction of cylindrical ferrogels placed on an electromagnet at different intensities of magnetic field were obtained experimentally depending on the concentration of magnetic particles in the samples. The experimental results showed that the attractive force increased with the intensity of the magnetic field, and with an increase in the concentration of magnetic particles in the FGs.

The experimental results were used to introduce a theoretical model based on the principal laws of magnetism to quantify the obtained relations. The model considered FG as a continuous medium with a magnetic susceptibility proportional to the volume fraction of the load of the magnetic particles irrespective of their distribution in the FG, which typically is quite uncertain in actual applications. Magnetic interactions between particles inside the FG were neglected. The intensity of a non-uniform magnetic field was taken as an average value along the distance from the magnet.

It was shown, that the developed model adequately predicted the mechanical force that emerged in ferrogel samples placed in a non-uniform magnetic field of an electromagnet. Despite the simplifications implied and the uncertainties neglected, the evaluation of the force occurred surprisingly fair—within the limits of the experimental error for the force measurements.

The external magnetic field can provide both the transportation force of an agent to the tissues of hollow organs and the controlled release of a drug from the macroporous ferrogel structure due to its mechanical deformation. The modeling of the mechanical force emerged in a ferrogel via an external field might be useful in platforms for cell proliferation and as implants for tissue replacement therapy [22,23,24]. In these cases, the external magnetic field can provide the controlled deformation of a ferrogel matrix which may trigger adhesion, proliferation, and differentiation of cells on a magnetically active platform [52]. Wound healing using ferrogel implants under the control of an external magnetic field is the most obvious biomedical application for the results of this study.

## Figures and Tables

**Table 1:** Measurements of force of attraction in FG samples under study at different strengths of magnetic field. Mean values (*n* = 3) and SD are displayed.

Field Intensity *H* (kA/m)	MPs—4.3% *F* (mN)	MPs—6.5% *F* (mN)	MPs—16.7% *F* (mN)	MPs—23.8% *F* (mN)
8.0	0.14 ± 0.08	0.34 ± 0.07	0.8 ± 0.2	1.6 ± 0.9
15.9	0.5 ± 0.1	1.1 ± 0.1	2.7 ± 0.7	5 ± 1
23.9	1.1 ± 0.2	2.6 ± 0.5	6 ± 2	11 ± 2
31.8	2.1 ± 0.6	4.5 ± 0.8	10 ± 2	19 ± 3
39.8	3.3 ± 0.4	7.4 ± 0.7	18 ± 2	28 ± 4

**Table 2:** Parameters related to the calculation of the susceptibility of the ferrogel samples.

Sample	Magnetite Weight Fraction (%)	Magnetite Volume Fraction (%)	Demagnetization Factor, *N*	Susceptibility, *χ*	M–H Slope $\frac{\chi }{1+N\chi }$
Epoxy composite	30.0	9.57	1/3	0.408	0.359
Ferrogel	23.8	6.36	0.43	0.254	0.229
Ferrogel	16.7	4.18	0.43	0.162	0.151
Ferrogel	6.5	1.49	0.43	0.056	0.054
Ferrogel	4.3	0.97	0.43	0.036	0.035
